# Exploring COVID-19-Associated Ileus: A Compelling Case Study

**DOI:** 10.7759/cureus.69647

**Published:** 2024-09-18

**Authors:** Harpreet Singh, Satnam Singh, Birkaran Sadhar, Brad Winegarden, Shawna Morrissey

**Affiliations:** 1 Medicine, Lake Erie College of Osteopathic Medicine, Erie, USA; 2 Surgery, Conemaugh Memorial Medical Center, Johnstown, USA; 3 Trauma and Acute Care Surgery, Conemaugh Memorial Medical Center, Johnstown, USA

**Keywords:** covid-19, covid ileus, gastrointestinal ileus, global epidemiology, health public

## Abstract

Since the start of 2020, COVID-19 has profoundly impacted global health. Classically, the disease manifests with pulmonary symptoms such as cough, dyspnea, pneumonia, and respiratory distress. However, it is important to note that in addition to these pulmonary symptoms, COVID-19 can present with extrapulmonary symptoms. This case report involves an 82-year-old male who tested positive for the causative agent of COVID-19 - severe acute respiratory syndrome coronavirus 2 (SARS-CoV-2), whose presentation manifested with gastrointestinal symptoms in the form of colonic ileus. This report aims to highlight the pathophysiological mechanisms of COVID-19-associated ileus while also delving into the importance of timely intervention to prevent complications like intestinal perforation.

## Introduction

COVID-19 has had a massive global impact. According to the World Health Organization (WHO), 776,137,815 individuals globally have tested positive for COVID-19 as of September 2024 [1]. COVID-19 emerges from infection with severe acute respiratory syndrome coronavirus 2 (SARS-CoV-2), a virus primarily targeting the respiratory system. The respiratory symptoms manifest heterogeneously. Some individuals can experience mild symptoms while others can experience acute respiratory distress [2]. Although COVID-19 is mostly associated with respiratory symptoms, it can lead to various extrapulmonary symptoms [3].

COVID-19 has been shown to manifest in thrombotic complications, myocardial dysfunction and arrhythmia, acute coronary syndromes, acute kidney injury, gastrointestinal symptoms, hepatocellular injury, hyperglycemia, ketosis, neurologic illnesses, ocular symptoms, and dermatologic complications [3]. The spike protein component of SARS-CoV-2 attaches to angiotensin-converting enzyme 2 (ACE-2) receptors on cell surfaces, facilitating cellular entry and infection. ACE-2 receptors are present in various extrapulmonary tissues, potentially accounting for the observed extrapulmonary symptoms. Additionally, endothelial damage and inflammation along ACE-2 pathways contribute to these symptoms [3]. The patient in this case experienced extrapulmonary manifestations.

The patient complained of abdominal pain, abdominal distension, nausea, and vomiting, which was shown to be colonic ileus. Gastrointestinal symptoms are often reported in COVID-19 patients. In a meta-analysis of 60 studies including 4243 patients, gastrointestinal symptoms occurred in 17.6% of patients. The most common symptoms reported were anorexia, diarrhea, nausea/vomiting, and abdominal pain. It is important to note that in 4-20% of cases, patients present with only gastrointestinal symptoms [4]. Furthermore, it has been noted that patients who only experience gastrointestinal symptoms may take longer to present to the hospital after their symptoms first appear [5]. This case report delves into an instance of a patient with COVID-19 who presented with gastrointestinal symptoms, specifically colonic ileus.

## Case presentation

**Figure 1:**
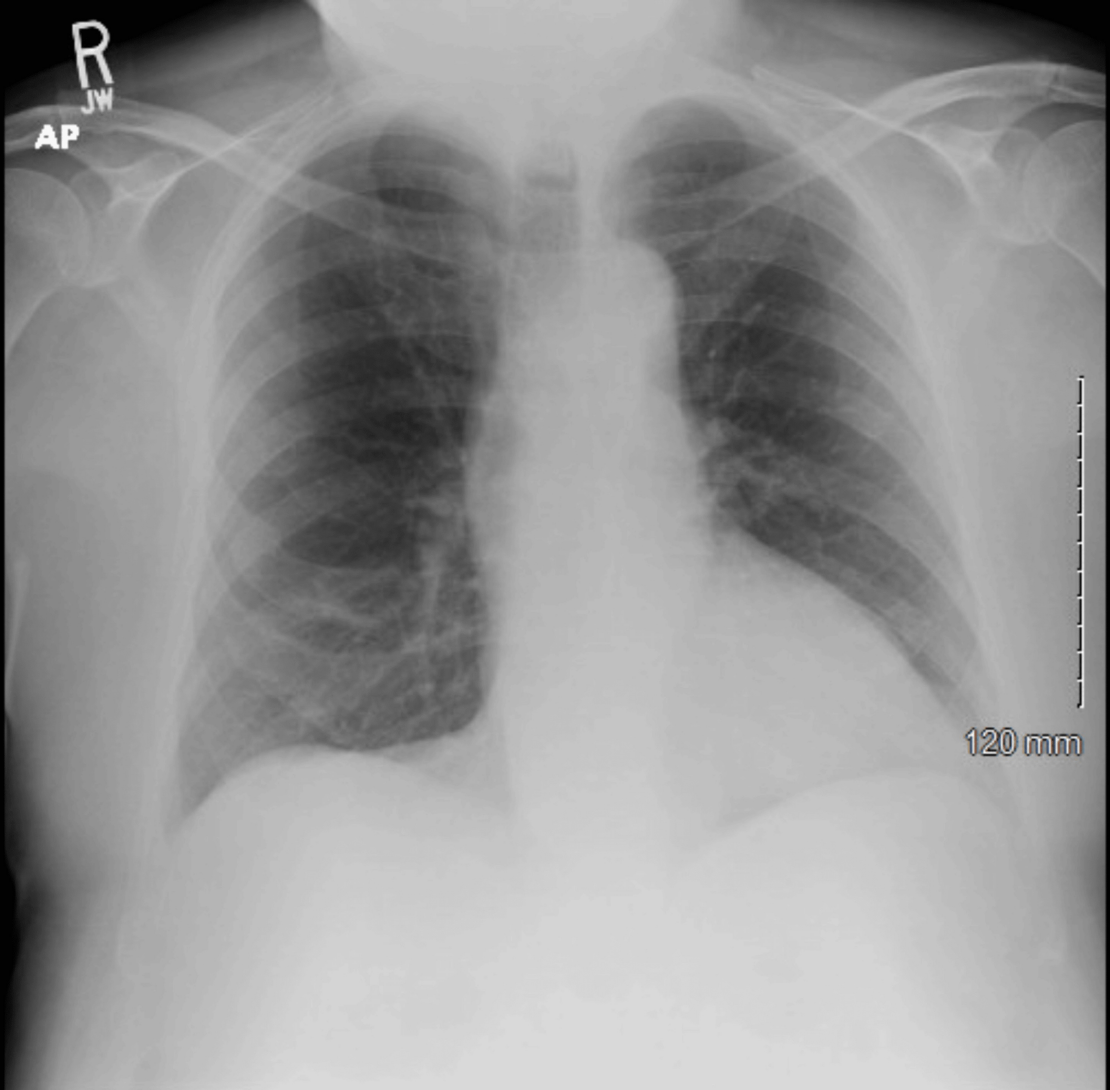
Normal chest X-ray

An 82-year-old male presented to the emergency department (ED) complaining of generalized weakness for two days. In the ED, the patient's blood pressure was initially 82/54. After receiving 1000 cc of normal saline, it improved to 92/58. The patient was found to have rhabdomyolysis with myoglobin greater than 20,000 ng/mL and creatine kinase (CK) greater than 7800 U/L. Transaminitis was seen with an aspartate transaminase (AST) of 1600 U/L and an alanine transaminase (ALT) of 300 U/L. The patient tested positive for COVID-19 while in the ED. Although the patient tested positive for COVID-19, the patient did not display any signs of respiratory compromise. The patient’s initial chest X-ray performed in the ED did not show any acute abnormality of the chest or any acute cardiopulmonary findings (Figure 1). While in the ED, the patient's systolic blood pressure dropped to 70. The patient was diagnosed with sepsis and was given broad-spectrum antibiotics of vancomycin and piperacillin-tazobactam. The patient was admitted to the intensive care unit (ICU) to receive vasopressors.

**Figure 2:**
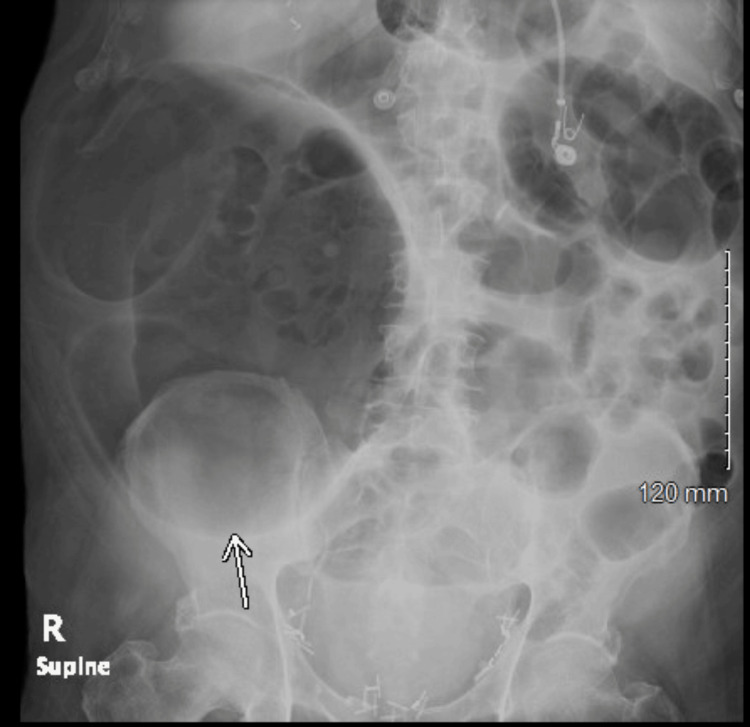
Abdominal X-ray showing dilation of the cecum (white arrow)

**Figure 3:**
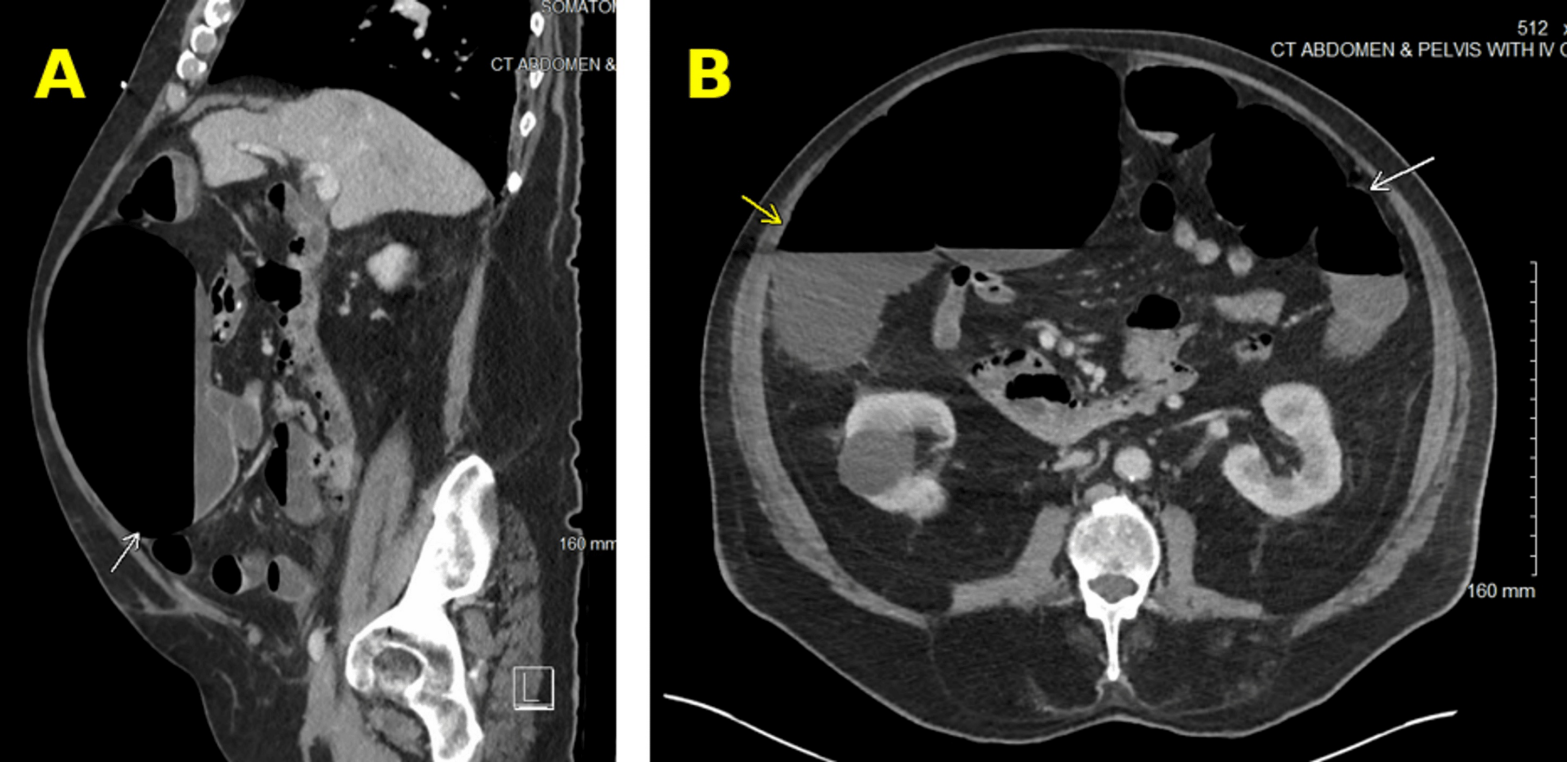
(A) Sagittal view of CT abdomen and pelvis showing cecal dilation (white arrow); (B) Axial view of CT abdomen and pelvis showing cecal dilation (yellow arrow) and fluid levels in the distal colon (white arrow)

While in the ICU, he received dexamethasone and methylprednisone for COVID-19. It was suspected that his hypotension, acute kidney injury on chronic kidney disease, and elevated transaminases were due to rhabdomyolysis. They also suspected rhabdomyolysis may be due to a recent increase in pravastatin dosing in the setting of McArdle disease. Antibiotics were stopped since hypotension was suspected to be from rhabdomyolysis and not septic shock. The patient was then weaned off the vasopressors and his condition was stable enough to be transferred to the general medicine floor. The patient had experienced nausea and vomiting after a meal on his sixth day in the hospital. His stomach was noted to be distended, and an X-ray was performed, which showed a dilated cecum (Figure 2). A CT scan was then performed, which showed colonic ileus (Figure 3). The CT showed dilation of the cecum up to 10 cm, fluid levels in the proximal and distal colon, and no signs of mechanical obstruction.

**Figure 4:**
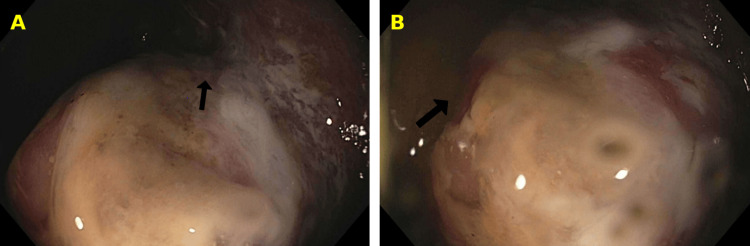
Images from colonoscopy: (A) shows inflammation of the ascending colon (black arrow); (B) shows inflammation of the cecum (black arrow).

Upon recognition of the distension and the patient’s inability to pass stool, the patient was given 1 mg IV neostigmine, which slightly improved the distension. After administration of the neostigmine, the patient reported being able to produce large bowel movements. The patient was still experiencing stomach distension so a nasogastric tube was placed with a total output of 400 cc of green bile. The patient removed the nasogastric tube citing discomfort. During physical examination, notable findings included pronounced abdominal distension, accompanied by hypoactive bowel sounds, firmness, and tympanic resonance upon percussion. The patient received three enemas that returned three liquid stools. The patient was not passing any flatus at this point. Gastroenterology was consulted and they performed a colonoscopy. The colonoscopy showed diffuse severe inflammation in the ascending colon secondary to ischemic colitis, dilation in the transverse colon and in the ascending colon that was decompressed, and a normal left side of the colon (Figure 4).

**Figure 5:**
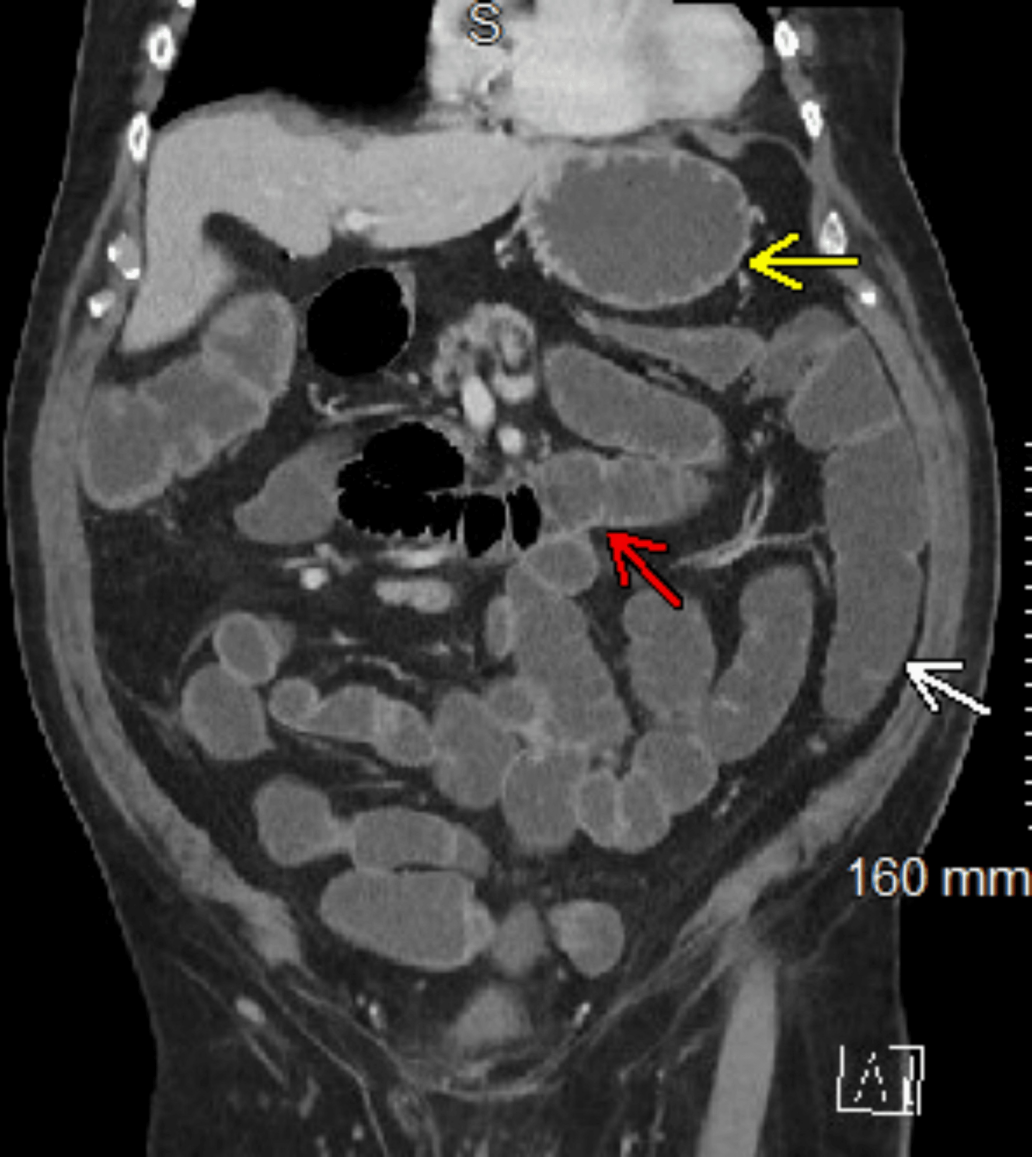
CT abdomen and pelvis showing fluid and gas distension of the stomach (yellow arrow), small intestine (red arrow), and colon (white arrow)

The following day, the patient did not show significant improvement following the colonic decompression or the three trials of neostigmine. The physical exam showed continued abdominal distension although depressible. The abdomen was not tender to palpation; however, the patient still reported experiencing symptoms of nausea and vomiting. Gastroenterology cited further endoscopic evaluation as being contraindicated due to failed colonic decompression in a patient with known colonic ischemic changes with slightly worsening distension. Gastroenterology recommended repeat imaging and evaluation by surgery. Repeat imaging was performed. CT of the abdomen and pelvis with IV contrast showed: 1) marked fluid and gas distension of the stomach, entire small intestine, and colon; 2) the fluid and air distension of the stomach and small intestine was new compared to the prior CT imaging, suggesting that these findings may be secondary to bowel obstruction, severe gastroenteritis/colitis, or severe paralytic ileus (Figure 5). The patient’s family requested a transfer, as the patient had not improved, and a second opinion was requested. The patient was stable for transfer and was accepted at another hospital.

## Discussion

Although SARS-CoV-2 is primarily recognized as a respiratory virus, patients have also been reported to manifest gastrointestinal symptoms [6]. The exact pathophysiological mechanisms behind COVID-19-induced ileus remain unclear. However, some hypotheses have been proposed. It is postulated that the virus may directly infect the gastrointestinal tract through the ACE-2 receptor, which is abundantly expressed in the gastrointestinal epithelium [6]. The same ACE-2 receptor is also present in the lungs and is responsible for facilitating the entry of the virus into the cells. In the epithelial cells of the gastrointestinal tract, ACE-2 plays a crucial role in regulating amino acid balance, facilitating the expression of antimicrobial peptides, and maintaining the balance of the gut microbiome [7]. The disruption of these processes can alter the gut microbiome, which can lead to gut dysbiosis [7]. In a study done in Hong Kong, the effects of gut microbiome alterations were studied. The study showed an inverse correlation between COVID-19 severity and the abundance of *Faecalibacterium prausnitzii, *an anti-inflammatory bacterium, in the gut microbiome [7]. This is one of the possible explanations as to how SARS-CoV-2 can infect the enteric nervous system, thus disrupting gut motility [8]. Furthermore, it is important to note that this gut dysbiosis can lead to increased cytokine levels, systemic inflammation, and an exaggerated immune response [7].

The exaggerated immune response in COVID-19 can trigger a cytokine storm by dysregulating the immune system, leading to an uncontrolled release of cytokines, which are signaling proteins that regulate immune responses. A cytokine storm is an excessive immune response characterized by the overproduction of pro-inflammatory cytokines, which can lead to severe tissue damage and organ failure. In the context of COVID-19, the cytokine storm involves key cytokines such as tumor necrosis factor-alpha (TNF-α), interleukin 6 (IL-6), and interleukin 1 (IL-1). These cytokines are central to the hyperinflammatory state that can lead to severe complications such as acute respiratory distress syndrome and multiorgan failure. As a result, these mechanisms are currently being explored to mitigate the effects of the cytokine storm [9]. It is imperative to treat COVID-19 infection early, as 14% of patients progress to severe disease and 5% to critical illness [10].

Possible treatments to mitigate the effects of a cytokine storm include the use of IL-6 inhibitors like tocilizumab, which aim to reduce the levels of pro-inflammatory cytokines and thereby alleviate the severity of the disease. Furthermore, corticosteroids and programmed cell death protein 1 (PD-1) checkpoint inhibitors have also been explored to combat the cytokine storm. Tocilizumab, a recombinant humanized monoclonal antibody targeting the IL-6 receptor, demonstrated in a retrospective study involving 21 individuals an ability to rapidly control severe COVID-19 symptoms, including fever and respiratory dysfunction [10]. Corticosteroids are currently shown to not be effective, as some studies have shown an increase in mortality. Currently, corticosteroids to counteract the cytokine storm are theorized as being ineffective; however, it remains an area requiring further exploration [10]. Lastly, PD-1 checkpoint inhibitors are currently being explored to reverse the anergy of lymphocytes that occur in COVID-19. Unfortunately, no study has been shown in the treatment of COVID-19. While targeting cytokine production appears logical, it's important to recognize that these cytokines are initially generated by the immune system to clear the virus. Consequently, suppressing the cytokine storm in its early stages remains controversial and warrants further research [10].

Lastly, in cases of ileus, electrolyte imbalances, and medication side effects should be ruled out. Metabolic ileus can occur in patients with diabetes mellitus or hypokalemia. The patient’s electrolyte values were within normal limits, and the patient was not diabetic. Ileus is a well-documented medication side effect that has been commonly associated with opioids and neuroleptic drugs [11]. The patient was not on any of these medications, making medication-induced ileus an unlikely etiology.

## Conclusions

This case report adds to the growing recognition of gastrointestinal manifestations in COVID-19 patients, specifically the occurrence of colonic ileus. The patient's presentation with abdominal distension, nausea, and vomiting, without the hallmark respiratory symptoms of COVID-19, emphasizes the importance of considering extrapulmonary complications in COVID-19 management. Our case underscores the need for early recognition of gastrointestinal involvement, as delayed diagnosis could lead to severe complications such as bowel ischemia or perforation. Timely intervention plays a critical role in managing these cases effectively.

The findings in this case suggest that the pathophysiology of COVID-19-associated ileus may involve the ACE-2 receptor-mediated viral invasion of the gastrointestinal tract, gut dysbiosis, and the cytokine storm, all contributing to impaired gut motility. This case highlights the importance of interdisciplinary care and a high index of suspicion for gastrointestinal complications in COVID-19, which may otherwise go unrecognized. Further research is warranted to clarify the mechanisms behind COVID-19-related ileus and to develop targeted therapeutic approaches.
